# Immunoregulatory Monocyte Subset Promotes Metastasis Associated With Therapeutic Intervention for Primary Tumor

**DOI:** 10.3389/fimmu.2021.663115

**Published:** 2021-06-07

**Authors:** Takumi Shibuya, Asami Kamiyama, Hirotaka Sawada, Kenta Kikuchi, Mayu Maruyama, Rie Sawado, Naoki Ikeda, Kenichi Asano, Daisuke Kurotaki, Tomohiko Tamura, Atsuko Yoneda, Keisuke Imada, Takashi Satoh, Shizuo Akira, Masato Tanaka, Satoshi Yotsumoto

**Affiliations:** ^1^ Laboratory of Immune Regulation, Tokyo University of Pharmacy and Life Sciences, Hachioji, Japan; ^2^ Department of Immunology, Yokohama City University Graduate School of Medicine, Yokohama, Japan; ^3^ Advanced Medical Research Center, Yokohama City University, Yokohama, Japan; ^4^ Laboratory of Genome and Biosignals, Tokyo University of Pharmacy and Life Sciences, Hachioji, Japan; ^5^ Center for Fundamental Laboratory Education, School of Pharmacy, Tokyo University of Pharmacy and Life Sciences, Hachioji, Japan; ^6^ Department of Immune Regulation, Graduate School and Faculty of Medicine, Tokyo Medical and Dental University (TMDU), Tokyo, Japan; ^7^ Laboratory of Host Defense, WPI Immunology Frontier Research Center (IFReC), Osaka University, Osaka, Japan

**Keywords:** surgery, irradiation, inflammation, atypical monocyte, lung metastasis

## Abstract

Systemic and local inflammation associated with therapeutic intervention of primary tumor occasionally promotes metastatic recurrence in mouse and human. However, it remains unclear what types of immune cells are involved in this process. Here, we found that the tissue-repair-promoting Ym1^+^Ly6C^hi^ monocyte subset expanded as a result of systemic and local inflammation induced by intravenous injection of lipopolysaccharide or resection of primary tumor and promoted lung metastasis originating from circulating tumor cells (CTCs). Deletion of this subset suppressed metastasis induced by the inflammation. Furthermore, transfer of Ym1^+^Ly6C^hi^ monocytes into naïve mice promoted lung metastasis in the mice. Ym1^+^Ly6C^hi^ monocytes highly expressed matrix metalloproteinase-9 (MMP-9) and CXCR4. MMP-9 inhibitor and CXCR4 antagonist decreased Ym1^+^Ly6C^hi^-monocyte-promoted lung metastasis. These findings indicate that Ym1^+^Ly6C^hi^ monocytes are therapeutic target cells for metastasis originating from CTCs associated with systemic and local inflammation. In addition, these findings provide a novel predictive cellular biomarker for metastatic recurrence after intervention for primary tumor.

## Introduction

Systemic and local inflammation caused by cancer therapy is now recognized as an important risk factor for cancer recurrence. Surgical resection of primary tumor, chemotherapy, or radiation therapy can awake dormant cancer cells and induce metastatic outgrowth in distant organs through inflammation (1–7). In addition to these cancer treatments, it has also been reported that inflammation caused by bacterial infection and cigarette smoke-exposure, promotes cancer dormancy escape and metastasis (8, 9). Such immune cells as neutrophils, macrophages, and monocytes are involved in cancer recurrence caused by inflammation. Recently, neutrophils have received increased attention with regard to their role in promoting cancer progression and metastasis associated with inflammation. For instance, neutrophils were reported to play critical roles in promoting lung metastases mediated by producing proinflammatory cytokines (10). Neutrophil extracellular traps (NETs) awake dormant cancer cells through interaction with cancer cells. NETs also trap circulating tumor cells (CTCs) and lead to increased formation of metastasis (9, 11, 12). In addition to neutrophils, monocytes and macrophages were also reported to be involved in cancer recurrence. The depletion of CD11b^+^ macrophages reduces lung metastasis of breast cancer cells (13). Vascular endothelial growth factor A (VEGFA)-secreting macrophages promote the extravasation of cancer cells and lung metastasis (14). It was also reported that monocytes recruited to metastasis site by the CCL2-CCR2 axis differentiate into macrophages and promote extravasation and survival of cancer cells (14, 15). A recent report indicated that not only neutrophils, but also monocytes, awake dormant cancer cells (7). Considering these reports, the types of immune cells involved in cancer progression and metastasis presumably depend on the context of inflammation or experimental models. However, immune cells involved in actual cancer-related events in patients are not well understood.

Blood monocytes play critical roles in inflammation as a component of mononuclear phagocyte system. In the steady-state conditions, monocytes consist of two or three subpopulations in mouse or human, respectively (16, 17). Classical monocytes (Ly6C^hi^CCR2^+^CX3CR1^−^ in mouse, CD14^+^CD16^−^ in human) are recruited into an inflamed site in a CCR2-dependent manner, and act as inflammation-promoting immune cells (14, 18, 19). In contrast, non-classical monocytes (Ly6C^low^CCR2^−^CX3CR1^+^ in mouse, CD14^dim^CD16^+^ in human) are differentiated from Ly6C^hi^ monocytes in an Nr4A-dependent manner, patrol the vasculature during homeostasis, and contribute to cancer immunosurveillance (20). Intermediate dim → + monocytes (CD14^+^CD16^+^) in human have been suggested to be responsible for the proliferation and stimulation of T cells (21). These monocyte subsets have been considered to coordinately engage in various immune responses in tissue injury or cancer. Recently, however, emergency hematopoiesis including monopoiesis during inflammation or other immune responses has been extensively studied, and several reports have identified bone marrow (BM)-derived atypical novel monocyte subsets that are rarely observed in the steady-state condition. In mouse, inflammation induced by microbial stimulation gives arise to neutrophil-like Ly6C^hi^ monocytes derived from granulocyte-macrophage progenitors (GMPs), but not MDPs (22, 23). Ceacam1^+^Msr^+^Ly6C^low^ monocytes called segregated-nucleus-containing atypical monocytes (SatM) emerge in lung of bleomycin-treated mouse and are involved in fibrosis (24). Ly6C^hi^MHCII^hi^Sca-1^hi^ monocytes arise in BM of acute gastrointestinal infected mouse and are considered to regulate immune response *via* the production of prostaglandin E2 and IL-10 (25). These reports suggest the possibility that a novel inflammation-related subset of monocytes can modulate cancer progression and metastasis associated with inflammation. However, details of such a monocyte subpopulation remain unknown.

We previously reported that GMP-derived atypical Ly6C^hi^ monocytes characterized by Ym1 expression (Ly6C^hi^Ym1^+^ monocytes) are produced in BM during the recovery phase of tissue injury. These monocytes share some characteristics with granulocytes and exhibit the immunoregulatory phenotype that contributes to tissue repair and regeneration (22). Here, we show that not neutrophils, but Ym1^+^Ly6C^hi^ monocytes contribute to promoting metastasis caused by inflammation associated with intervention for primary tumor. These findings demonstrate that the mechanisms of tissue repair are closely related to metastasis and provide a novel therapeutic target for the metastasis.

## Methods

### Mice

C57BL/6J mice were obtained from CLEA Japan, Inc. CD204-DTR knock-in mice (26), Ym1-DTR knock-in mice, Ym1-Venus mice, and Lcn2 ^-/-^ mice were described previously (22, 27). All experiments using the mice described herein were approved by the Tokyo University of Pharmacy and Life Sciences Animal Use Committee (L18-22, L18-23, L19-20, L19-21, L20-17, and L20-18) and performed in accordance with applicable guidelines and regulations.

### Reagents

For the induction of inflammation, lipopolysaccharides (LPS; *E. coli*, O111:B4) (Sigma), CpG-ODN (ODN1668; Hokkaido System Science), and Poly(I:C) (GE Healthcare Life Sciences) were used. For the depletion of monocytes and/or neutrophils, anti-Gr-1 (clone RB6-8C5, in-house purification) or anti-Ly6G (clone 1A8; BioXCell) was used. For the inhibition of MMP-9 activity and CXCR4, SB-3CT (Tokyo Chemical Industry) and AMD3100 (Sigma) were used respectively. Diphtheria toxin (DT) was purchased from Sigma. For the detection of IL-6 and TNF-alpha concentrations in serum, an ELISA MAX™ Standard Set was purchased from BioLegend. For analysis of cell surface marker expression, the following Abs were used: anti-CD11b-PE (clone M1/70), anti-CD62L-PE (clone MEL-14), anti-F4/80-PE (clone RM8), anti-C5aR-PE (clone 20/70), anti-MHC-II-PE (clone M5.114.15.2), anti-VCAM1-PE [clone 429 (MVCAM)], anti-Ly6G-PE (clone 1A8), anti-CXCR4-APC (L276F12), and anti-Treml4-PE (clone 16E5) were purchased from BioLegend. Anti-PD-L1-PE (clone MIH5) was purchased from Thermo Fisher Scientific. Anti-CD204-PE (clone REA148) was purchased from Miltenyi Biotec. Anti-CXCR2-APC (clone 242216) and anti-CCR2-APC (clone 475301R) were purchased from R&D Systems. Anti-CD131-PE (clone JORO50) was purchaced from BD Biosciences.

### Cell Lines

The murine melanoma cell line, B16F10 (Riken Cell Bank, Ibaraki, Japan), was maintained in RPMI1640 medium supplemented with 10% fetal bovine serum (FBS) and 100 units/mL of penicillin-streptomycin at 37°C in a humidified incubator with 5% CO_2_.

### Preparation of Cells

BM monocytes were isolated by cell sorter or Monocyte Isolation Kit (#130-100-629 Miltenyi Biotech). For BM monocyte isolation using cell sorter, BM cells from WT- or Ym1-Venus mice were incubated with anti-CD16/32 (clone 93) and then with a cocktail of biotinylated-anti-Lin [CD4 (Clone GK1.5), CD8 (Clone 53-6.7), B220 (RA3-6B2), NK1.1 (Clone PK136), Ly6G (Clone 1A8) and Ter119 (Clone TER-119)] antibodies in MACS buffer (phosphate-buffered saline (PBS), pH 7.2; 2 mM EDTA; 0.5% bovine serum albumin), followed by incubation with anti-biotin microbeads (#130-090-485 Miltenyi Biotech). Lin^+^ cells were depleted by magnetic sorting (autoMACS Pro Separator, Miltenyi Biotech). Lin^-^ cells were stained with anti-CD45.2-PE-Cy7 (BioLegend, clone 104), anti-Ly6G-APC (BioLegend, clone 1A8), anti-CD115-Brilliant Violet 421 (BioLegend, clone AFS98) and anti-Ly6C-PE (BioLegend, clone HK1.4) antibodies and then fractionated by a cell sorter (SH800, SONY, or AriaIII, BD Biosciences). For the analysis of the number of monocytes and tumor cells in lung, sorted monocytes and B16 cells were stained with PKH-26 (red fluorescence) and PKH-67 (green fluorescence) (Sigma), respectively, according to the manufacture’s protocol. For the isolation of lung cells, lungs were fragmented and transferred to a conical tube containing digestion solution (0.2 U/mL Liberase TL (#5401020001, Roche), 1 µg/mL DNase I (#DN25, Sigma) in HBSS). Samples were incubated at 37°C under agitation for 25 min. After incubation, the cells were dispersed by pipetting and pelleted by centrifugation. The cells were then washed with MACS buffer. To deplete erythrocytes, the cells were treated with BD Pharm Lyse™ - Lysing Buffer (BD Biosciences) and then washed with MACS buffer. For the analysis of peripheral blood mononuclear cells, peripheral blood was collected in an EDTA-containing tube. Then, the red blood cells were lysed with BD Pharm Lyse™ - Lysing Buffer.

### Experimental Metastasis Assay

B16 cells (1 × 10^5^ cells) were injected intravenously into WT-, CD204-DTR-, or Ym1-DTR mice to generate lung metastases. The number of nodules reflecting lung metastasis of B16 was visually counted. To evaluate melanoma-related mRNA expression in lung, total RNA from snap-frozen-lung tissue was extracted with a FavorPrep Total RNA Extraction Column (Favorgen) according to the manufacturer’s protocol. For qRT-PCR, cDNAs were synthesized using ReverTra Ace (TOYOBO). qRT-PCR was performed on cDNA with a THUNDERBIRD SYBR qPCR Mix (TOYOBO). Expression levels were normalized to 18s ribosomal RNA (rRNA). The following primer sequences were used for each gene: *Pmel* forward 5’-GCTTGTAGGTATCTTGCTGGTGTT-3’, reverse 5’-CCTGCTTCTTAAGTCTATGCCTATG-3’; *Dct* forward 5’-GGCTACAATTACGCCGTTG-3’, reverse 5’-CACTGAGAGAGTTGTGGACCAA-3’; and *18s rRNA* forward 5’-CGGACAGGATTGACAGATTG-3’, reverse 5’-CAAATCGCTCCACCAACTAA-3’. For experimental metastasis assay with tumor resection, 1 × 10^6^ B16 cells were implanted subcutaneously into the back of WT mice. Six or seven days after implantation, mice under anesthesia underwent tumor tissue resection through cutaneous incision. Twenty-four hours later, B16 cells (1 × 10^5^ cells) were injected intravenously to generate lung metastases. Lung metastasis of B16 was estimated as above.

### X-Ray Irradiation

B16 cells (1 × 10^6^ cells) were implanted subcutaneously into the back of WT mice. Seven to eight days after implantation, mice under anesthesia were immobilized in a customized harness that allowed the implanted tumor to be exposed, whereas the remainder of the body was shielded by 3.5 cm of lead. Mice were irradiated in a Faxitron CP-160 irradiator (Faxitron X-ray Corporation).

### Quantitative RT-PCR (qRT-PCR)

For the analysis of mRNA levels in Ym1^+^- or Ym1^-^ Mo, sorted Ym1^+^- or Ym1^-^ Mo RNA was extracted and converted into cDNA, and qRT-PCR was performed on the cDNA as above. Expression levels were normalized to 18s rRNA. The following primer sequences were used for each gene: *Chi3l3* forward 5’-AAAGACAAGAACACTGAGCTAAAAACTC-3’, reverse 5’-GAATCTGATAACTGACTGAATGAATATC-3’; *MMP-9* forward 5’-CTTCCCCAAAGACCTGAAAAC-3’, reverse 5’-CTGCTTCTCTCCCATCATCTG-3’; *Il1b* forward 5’-GGATGAGGACATGAGCACCT-3’, reverse 5’-AGCTCATATGGGTCCGACAG-3’; *Vegfa* forward 5’-AAAAACGAAAGCGCAAGAAA-3’, reverse 5’-TTTCTCCGCTCTGAACAAGG-3’; *Cox2* forward 5’-CCAGCACTTCACCCATCAGTTTTTCAAG-3’, reverse 5’-CAGTTTATGTTGTCTGTCCAGAGTTTCA-3’; and *Lcn2* forward 5’-CCATCTATGAGCTACAAGAGAACAAT-3’, reverse 5’-TCTGATCCAGTAGCGACAGC-3’.

### RNA-Sequencing

Sorted cells were lysed and their total RNAs were extracted with RNeasy Mini kit (QIAGEN). Five hundred picograms of total RNA was subjected to DNA library preparation for RNA sequencing analysis using SMART-Seq v4 Ultra Low Input RNA Kit (TAKARA) and Nextera XT DNA Library Preparation Kit (Illumina). Sequencing was performed on a NextSeq 500 sequencer (Illumina) in the 75-bp single-end read mode. Data with the fragments per kilobase of exon per million reads (FPKM) were used for further analysis after mapping of the sequence reads. PCA analysis of RNA-sequencing was performed using AltAnalyze. The R package limma was used to identify differentially expressed genes. For PCA analysis, RNA-seq data in BM naïve monocytes, Ym1^+^Ly6C^hi^ monocytes, and Ym1^-^Ly6C^hi^ monocytes were retrieved from the Gene Expression Omnibus database (accession number GSE118032) (22).

### Western Blotting

Lungs were thoroughly homogenized in a homogenizer (Bioprep-6, Allsheng, Hangzhou, China) at 3800 rpm for four cycles, and 0.2 s per cycle, in RIPA buffer (50 mM Tris HCl [pH 7.4], 1% NP-40, 0.5% sodium deoxycholate, 0.1% SDS, 150 mM NaCl) with protease inhibitors (#11836145001, Roche). For 10 mg of tissue, 500 μL of RIPA buffer was used. After 30 min on ice, the samples were centrifuged at 10,000 × g for 20 min at 4°C, and protein concentration in the supernatant was determined using the bicinchoninic acid (BCA) protein assay (#23225, Thermo Fisher Scientific). Equal amounts of protein from each sample were loaded on SDS-polyacrylamide gel electrophoresed, separated, and transferred onto PVDF membranes. The immunoblots were incubated in blocking buffer [5% skim milk in phosphate-buffered saline (PBS) with 0.1% Tween 20 (PBST)] for 60 min at room temperature and probed with anti-citrullinated histone H3 (#ab5103, Abcam) or anti-GAPDH mAb-HRP-DirecT (#M171-7, Medical & Biological Laboratories) overnight at 4°C. Then, the immunoblots were washed three times for 5 min in PBST, incubated with polyclonal goat anti-rabbit IgG-HRP (#P0448, Dako) for 30 min at room temperature in blocking buffer, and washed three times in PBST again. Immunodetection was performed using a SuperSignal™ West Pico PLUS Chemiluminescent Substrate (#34580, Thermo Fisher Scientific).

### Immunohistochemistry

Lungs were harvested and embedded in OCT compound (SECTION-LAB, Japan). The cut surface was covered with an adhesive film (Cryofilm type IIC9, SECTION-LAB, Japan) and frozen sections (5 µm) were prepared with a macrotome (CM3050S Leica Microsystems, Germany). The resulting sections were post-fixed with 100% EtOH for 10 s and 4% PFA/PBS(-) for 10 s, rinsed with PBS(-) for 20 s, and incubated with TNB Blocking Buffer [0.1 M Trizma Base, pH7.5, 0.15 M NaCl, 0.5% (w/v) blocking reagent (PerkinElmer, FP1020)] for 1 h at room temperature. The sections were then incubated with anti-citrullinated histone H3 antibody (1/250), or MPO antibody (#AF3667, R&D Systems, 1/100) in TNB Blocking Buffer for 1 h at room temperature. After three washes with PBS (-), the sections were incubated with donkey anti-rabbit IgG-Cy3 (#406402, Biolegend, 1/1000), or donkey anti-goat IgG-Alexa 488 (#705-545-003, Jackson ImmunoResearch, 1/1000) in TNB Blocking Buffer for 1 h in the dark at room temperature. After two washes with PBS(-) and one wash with water, the sections were counterstained with DAPI, and the slides were covered with cover slips using mounting media (FluorSave Reagent, 345789, Merck Millipore).

### Gelatin Zymography

A conditioned medium from monocytes (5 × 10^6^ cells/mL, in a 24-well plates containing Advanced RPMI1640 medium (Thermo Fisher Scientific), grown on plastic for 24 h), was mixed 4:1 ratio with loading buffer (0.125 M Tris, pH 6.8, 4% SDS, 20% glycerol, 0.01% bromophenol blue). Then, the samples were loaded on Novex™ 10% Zymogram Plus (Gelatin) Protein Gels (#ZY00102BOX, Thermo Fisher Scientific). After electrophoresis, the gels were rinsed twice with water and incubated in washing buffer (50 mM Tris, pH 7.5, 0.016% NaN_3_, 2.5% Triton X-100, 5 mM CaCl_2_, 1 µM ZnCl_2_) for 30 min at room temperature. Then, the gels were rinsed with incubation buffer (50 mM Tris, pH 7.5, 0.016% NaN_3_, 1% Triton X-100, 5 mM CaCl_2_, 1 µM ZnCl_2_) for 10 min at 37°C and incubated in incubation buffer at 37°C for 16 h. The gels were stained with 0.5% Coomassie Blue R-250 (#031-17922, Wako; diluted with 40% ethanol and 10% acetic acid) and destained with 40% ethanol and 10% acetic acid.

### Invasion Assay

Ly6C^hi^ monocytes (2 or 5 × 10^6^/mL) sorted from LPS-treated WT- or Ym1-Venus mice were incubated in serum-free medium (Advanced-RPMI1640, Thermo Fischer Scientific) at 37°C in 5% CO_2_ for 24 h. The culture supernatant was centrifugated (10,000 ×g, 30 min at 4°C). The supernatants were collected for the invasion assay. Human melanoma cell line A375 (ATCC-CRL-1619, 1 × 10^6^/mL) was suspended in serum-free medium (RPMI1640, Wako) and added into the upper chamber of a 24-well Transwell chamber that had been coated with Matrigel (Corning, BioCoat 354480) in the presence or absence of monocyte-culture supernatant. The lower chamber contained RPMI1640 containing 0.1% FBS as a chemoattractant. Assays were carried out at 37°C in 5% CO_2_ for 24 h. At the end of the incubation, the non-invading cells on the upper surface of the filter were mechanically removed. The invading cells that migrated through the Matrigel and the 8-µm pore membrane, were fixed with 4% paraformaldehyde/PBS for 5 min, and stained with 0.1% crystal violet (WAKO) for 20 min. The proportion of invading cells was calculated using BZ-X710 software (Keyence).

### Statistics

Data were analyzed either by analysis of variance (ANOVA) followed by multiple comparison, or by the t-test with Prism (GraphPad Software, CA). *P* values < 0.05 were considered significant.

## Results

### Ly6C^hi^ Monocytes, but Not Neutrophils, Promote Lung Metastasis Accelerated by Systemic Inflammation

**Figure 1:**
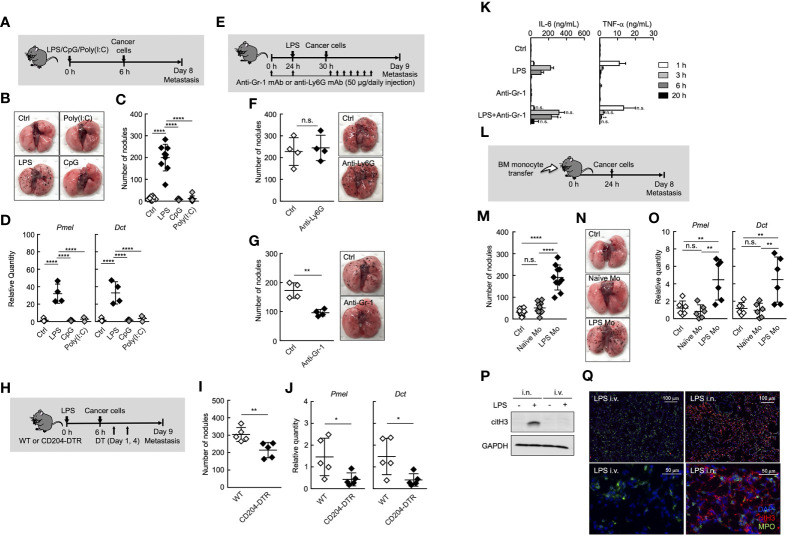
Ly6C^hi^ monocytes promote lung metastasis in systemic inflammatory state. **(A–D)** Effects of TLR ligands on lung metastasis. **(A)** Experimental design for analyzing the effect of TLR ligands on metastatic progression. WT mice were injected with either PBS (Ctrl), 20 µg of LPS, 100 µg of CpG-ODN (CpG), or 100 µg of Poly(I:C) followed by i.v. injection of B16 cells (1 x 10^5^ cells) 6 h later. The lungs were analyzed for metastasis on Day 8. **(B)** Representative images of lungs on Day 8. **(C)** Quantitative summary of the number of lung metastases on Day 8. **(D)** mRNA expression levels of B16 melanoma cell-specific genes were determined by qRT-PCR and are shown as fold change relative to control lungs. Average values are shown with SD. One-way ANOVA followed by Dunnett’s test, n = 5-9 **(C, D)**. ****P < 0.001; n.s., not significant. Each symbol represents an individual animal. **(E–G)** Effects of immune cell deletion on lung metastasis. **(E)** Experimental design used to test the effect of anti-Gr-1 mAb and anti-Ly6G mAb on metastatic progression. WT mice were injected with 20 µg of LPS on Day 1 followed by i.v. injection of B16 cells (1 x 10^5^ cells) 6 h later. For deletion of neutrophils alone (anti-Ly6G), or monocytes and neutrophils (anti-Gr-1), 50 µg/daily of indicated mAbs or PBS (Ctrl) were injected into these mice from Day 0 to Day 8. The lungs were analyzed for metastasis on Day 9. **(F, G)** Quantitative summary of the number of lung metastases (left), and representative images of the effect of the lungs metastasis (right). Average values are shown with SD. Unpaired two-tailed t-test, n = 4 **(F, G)**. **P < 0.01; n.s., not significant. Each symbol represents an individual animal. **(H–J)** Reduced number of lung metastases in CD204-DTR mice. **(H)** Experimental design used to test the contribution of CD204^+^ cells to metastatic progression. WT and CD204-DTR mice were injected intravenously with 20 µg of LPS. Six hours later, B16 cells were injected intravenously, and this was followed by the i.p. injection of DT (500 ng/injection) on Days 1 and 4. The lungs were analyzed for metastasis on Day 9. **(I)** Quantitative summary of the number of lung metastases on Day 9. **(J)** mRNA expression levels of indicated genes in lungs are shown as fold change relative to lung of DT-treated WT mice. Average values are shown with SD. Unpaired two-tailed t-test, n = 5 (J and K). **P < 0.01; *P < 0.05. Each symbol represents an individual animal. **(K)** WT mice were injected intraperitoneally (i.p.) with anti-Gr-1 mAb (50 µg/injection) at -24 and 0 h and intravenously with LPS at 0 h. Sera were collected at 1, 3, 6, and 20 h after LPS injection. Serum cytokine concentrations were measured by ELISA. Average values are shown with SD. Unpaired two-tailed t-test at each time point, compared with LPS injection, n = 3-4. **P < 0.01; *P < 0.05; n.s., not significant. **(L–O)** Increased number of lung metastases in LPS Mo-transferred mice. **(L)** Experimental design used to test the effects of monocyte transfer on metastatic progression. WT mice were transferred intravenously with advanced RPMI1640 (Ctrl) or Ly6C^hi^ monocytes prepared either from naïve mice (Naïve Mo) or LPS-treated mice (LPS Mo) (5 x 10^5^ cells). Twenty-four hours later, B16 cells were injected intravenously. The lungs were analyzed for metastasis on Day 8. **(M)** Quantitative summary of the number of lung metastases on Day 8. **(N)** Representative images of the lungs. **(O)** mRNA expression levels of indicated genes in lungs are shown as fold change relative to control lungs. Average values are shown with SD. One-way ANOVA followed by Dunnett’s test, n = 6–10 **(M, O)**. ****P < 0.001; **P < 0.01; n.s., not significant. Each symbol represents an individual animal. **(P, Q)** Intranasal (i.n.) but not i.v. injection of LPS induces NET formation in lung. WT mice were injected i.v. or i.n. with LPS (20 or 10 µg, respectively). Twenty-four hours later, the lungs were analyzed. **(P)** Western blot analysis for citrullination of histone H3 (citH3) in lungs of LPS-treated mice. Western blot analysis of lung tissues was performed as described in Materials and Methods. **(Q)** Immunohistochemistry of lung section from LPS-treated WT mice. Images show representative immunostaining of myeloperoxidase (MPO: green), citH3 (red), and DAPI (blue) in the lung of mice treated with LPS. Original magnification, ×20 (upper panel) and × 100 (lower panel). The data shown are representative of two independent experiments.

Inflammation is one of the most important factors that promote cancer metastasis (2, 7–11, 28–30). The metastasis cascade involves multiple processes, including invasion of cancer cells into adjacent tissue, intravasation, survival in blood circulation, extravasation of circulating tumor cells (CTCs), and subsequent outgrowth at distant sites (31). Among these steps, the outgrowth of CTCs at distant sites was proven to be enhanced by systemic inflammation in an experimental metastasis model (8, 10, 11, 29, 30, 32). However, the precise mechanisms of inflammation-induced metastasis originating from CTCs remain unknown. To explore these mechanisms, we first compared some forms of systemic inflammation induced by different Toll-like receptor (TLR) ligands from the perspective of promoting metastasis in an experimental metastasis model (**Figure 1A**). Mice were treated with different TLR ligands, followed by intravenous (i.v.) injection of B16 melanoma cells. Consistent with previous reports (8, 10, 30), the systemic injection of lipopolysaccharide (LPS) *via* tail vein promoted the formation of metastatic foci of B16 melanoma cells originating from CTCs in lung (**Figures 1B, C**). The mRNA expression levels of premelanosome protein (*Pmel)* and dopachrome tautomerase (*Dct)* genes, both of which are highly expressed in B16 melanoma cells (33), were significantly elevated in the lungs of LPS-treated mice (**Figure 1D**), indicating outgrowth of B16 melanoma cells in the lungs. On the other hand, CpG-ODN or Poly (I:C) had negligible effects on metastasis (**Figures 1B–D**), indicating that systemic-inflammation-induced enhancement of metastasis depends on the mode of inflammation.

It was reported that neutrophils and monocytes are involved in metastasis under inflammatory conditions (7, 9–11). In fact, both neutrophils and monocytes accumulated in lung in the early phase (Day1 to 2 after systemic injection of LPS) of inflammation (**Supplemental Figures 1A, B**). Therefore, we focused on the role of neutrophils and monocytes in the inflammation-induced promotion of metastasis. The depletion of neutrophils by anti-Ly6G monoclonal antibody (mAb) injection had no effects on lung metastasis (**Figures 1E, F**, and **Supplemental Figure 2**). On the other hand, anti-Gr-1 mAb, which depletes both neutrophils and Ly6C^hi^ monocytes, but not Ly6C^low^ monocytes, suppressed the metastatic formation (**Figure 1G** and **Supplemental Figure 2**), suggesting that Ly6C^hi^ monocytes, but not neutrophils, contributed to promoting metastasis induced by systemic injection of LPS. We previously reported that BM and peripheral blood monocytes highly expressed CD204, a class A scavenger receptor (26), and that both Ly6C^hi^ and Ly6C^low^ monocytes but not neutrophils were specifically deleted in peripheral blood by diphtheria toxin (DT) injection in CD204-DTR mice (26) (**Supplemental Figure 3**). In these mice, the number of metastatic foci was decreased by DT injection (**Figures 1H–J**), indicating that monocytes are responsible for the promotion of lung metastasis. The injection of anti-Gr-1 mAb did not increase inflammatory cytokine production induced by LPS (**Figure 1K**), indicating that the suppression of metastasis by anti-Gr-1 mAb is not attributed to the suppression of inflammatory cytokine production.

Albrengues et al. recently reported that neutrophil extracellular traps (NETs) play a critical role in the awakening of dormant cancer cells and the growth of metastatic lesions in lung, when mice were injected intranasally (i.n.) with LPS (9). Thus, we compared i.n. and i.v. routes of LPS administration in terms of NET formation in lung. As was previously reported, the i.n. injection of LPS induced the citrullination of histone H3, a specific marker for NET formation in lung. On the other hand, the injection of LPS *via* tail vein never induced NET formation in lung (**Figures 1P, Q**). These results strongly suggest that NET formation is not attributed to inflammation-induced promotion of metastasis in the case of systemic injection of LPS.

To further confirm the role of Ly6C^hi^ monocytes in systemic-inflammation-induced metastasis originating from CTCs, we purified Ly6C^hi^ monocytes from either naïve or LPS-injected mice and injected intravenously these monocytes into naïve mice. After that, we injected cancer cells (**Figure 1L**). As shown in **Figures 1M–O**, Ly6C^hi^ monocytes from LPS- injected mice (LPS Mo) facilitated the formation of metastatic foci in lungs, whereas Ly6C^hi^ monocytes from naïve mice (Naïve Mo) did not. We counted the number of transferred monocytes and B16 cells in lung soon after injection of these cells. However, there was no significant difference in the cell number of these cells in the lungs between Naïve Mo-transferred- and LPS Mo-transferred mice (**Supplemental Figure 4**), suggesting functional difference between Naïve Mo and LPS Mo in lung. Taken together, the systemic injection of LPS provides Ly6C^hi^ monocytes with the ability to promote metastasis.

### Ym1^+^Ly6C^hi^ Monocyte Subset Plays a Vital Role in Lung Metastasis

**Figure 2:**
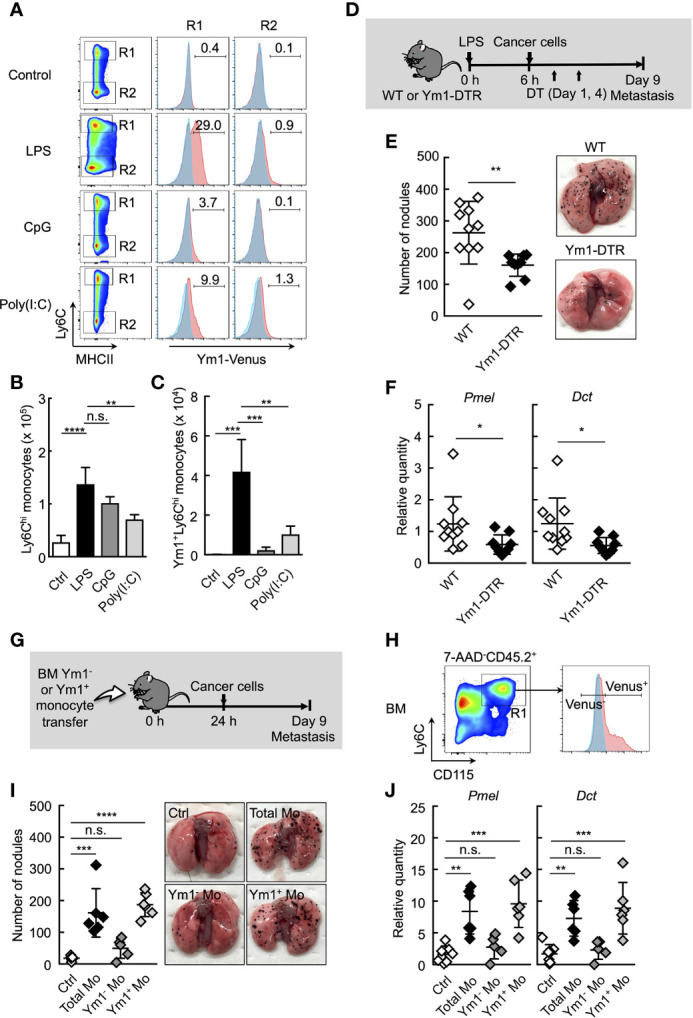
Ym1^+^Ly6C^hi^ monocyte subset plays a vital role in lung metastasis. **(A–C)** Flow cytometric analysis of the lung cells in WT- (shaded area in blue) or Ym1-Venus mice (in red). PBS (Ctrl), LPS, CpG, or Poly(I:C) was injected intravenously into WT- or Ym1-Venus mice. Forty-eight hours later, lung cells were stained for CD45.2, CD11c, CD11b, Ly6G, MHCII and Ly6C, and analyzed by flow cytometer as described in **Supplemental Figure 1A**. Numbers indicated percentage of Ym1^+^ cells in CD45.2^+^CD11c^-^CD11b^+^MHCII^-^Ly6C^hi^cells (R1; Ly6C^hi^ monocytes) or CD45.2^+^CD11c^-^CD11b^+^MHCII^-^Ly6C^low^cells (R2; Ly6C^low^ monocytes) **(A)**. Absolute numbers of Ly6C^hi^ monocytes **(B)** and Ym1^+^Ly6C^hi^ monocytes **(C)** in lungs. Average values are shown with SD. One-way ANOVA followed by Dunnett’s test, n = 3-5. ****P < 0.001; ***P < 0.005; **P < 0.01; n.s., not significant. **(D–F)** Reduced number of lung metastases in the absence of Ym1^+^Ly6C^hi^ monocytes. **(D)** Experimental design used to test the contribution of Ym1^+^ cells to metastatic progression. WT- and Ym1-DTR mice were injected intravenously with LPS. Six hours later, B16 cells were injected intravenously, and this was followed by the i.p. injection of DT (500 ng/injection) on Days 1 and 4. The lungs were analyzed for metastasis on Day 9. **(E)** Quantitative summary of the number of metastases in lungs (left), and representative images of lung metastasis (right). **(F)** mRNA expression levels of indicated genes in lungs are shown as fold change relative to lung of DT-treated WT mice. Average values are shown with SD. Unpaired two-tailed t-test, n = 9-10. **P < 0.01; *P < 0.05. Each symbol represents an individual animal. **(G–J)** Increased number of lung metastases in Ym1^+^Ly6C^hi^ monocyte-transferred mice. **(G)** Experimental design used to test the effects of transfer of Ym1^+^ or Ym1^-^ Ly6C^hi^ monocytes (Ym1^+^ Mo or Ym1^-^ Mo, respectively) on metastatic progression. Ym1^+^Ly6C^hi^ and Ym1^-^Ly6C^hi^ monocytes were sorted from BM of Ym1-Venus mice 48 h after LPS (20 µg) treatment. WT mice were transferred intravenously with advanced RPMI1640 medium (Ctrl), Ym1^+^ Mo or Ym1^-^ Mo (5 x 10^5^ cells). Twenty-four hours later, B16 cells (1 x 10^5^ cells) were injected intravenously. The lungs were analyzed for metastasis on Day 9. **(H)** Identification of Ym1^+^ Mo or Ym1^-^ Mo in BM for cell sorting. Samples were pregated on live CD45.2^+^ cells. **(I)** Quantitative summary of the number of lung metastases (left), and representative images of the lungs metastasis (right). **(J)** mRNA expression levels of indicated genes in lungs are shown as fold change relative to control lungs. Average values are shown with SD. One-way ANOVA followed by Dunnett’s test, n = 5-7 (I and J). ****P < 0.001; ***P < 0.005; **P < 0.01; n.s., not significant. Each symbol represents an individual animal.

We next sought to reveal the properties of Ly6C^hi^ monocytes in mice treated with LPS. We previously identified a subpopulation of Ly6C^hi^ monocytes that are characterized by a high expression of Ym1 (22). Ym1^+^Ly6C^hi^ monocytes greatly expanded in BM during the recovery phase of systemic inflammation induced by LPS administration or tissue injury. These monocytes infiltrating into an injured site exhibited immunoregulatory and tissue-reparative phenotypes. These findings of the roles of Ym1^+^Ly6C^hi^ monocytes in tissue repair prompted us to speculate that Ym1^+^Ly6C^hi^ monocytes could play roles in systemic inflammation-induced metastasis. We first monitored the accumulation of Ym1^+^Ly6C^hi^ monocytes in lung after systemic inflammation by using Ym1-Venus mice. As expected, when Ym1-Venus mice were injected with LPS, a large number of Ym1^+^Ly6C^hi^ monocytes were accumulated in the lungs (**Figures 2A–C**). Intriguingly, a small number of Ym1^+^Ly6C^hi^ monocytes were found in the lungs of mice injected with either CpG-ODN or Poly(I:C) (**Figures 2A–C**), both of which had no effects on metastasis formation in lung (**Figures 1B–D**). An increase in the number of Ym1^+^Ly6C^hi^ monocytes was also observed in mice injected with both LPS and cancer cells (**Supplemental Figures 5A, B**), suggesting the role of Ym1^+^Ly6C^hi^ monocytes in lung metastasis.

We previously generated Ym1-DTR mice in which Ym1-expressing cells were deleted by DT injection (**Supplemental Figure 6**). As shown in **Figures 2D–F**, the transient deletion of Ym1-positive cells on Days 1 and 4 significantly suppressed lung metastasis induced by LPS injection. To further reveal the role of Ym1^+^Ly6C^hi^ monocytes in promoting metastasis originating from CTCs, we purified Ym1^+^Ly6C^hi^ or Ym1^-^Ly6C^hi^ monocytes from BM of LPS-treated Ym1-Venus mice and injected those cells into naïve mice. After that, we injected cancer cells (**Figures 2G, H**). The injection of Ym1^+^Ly6C^hi^ monocytes resulted in a large number of metastatic foci in lung compared with the injection of Ym1^-^Ly6C^hi^ monocytes (**Figures 2I, J**). These results clearly indicate that Ym1^+^Ly6C^hi^ monocytes have the ability to promote lung metastasis.

### Ym1^+^Ly6C^hi^ Monocytes Express Metastasis-Related Genes

**Figure 3:**
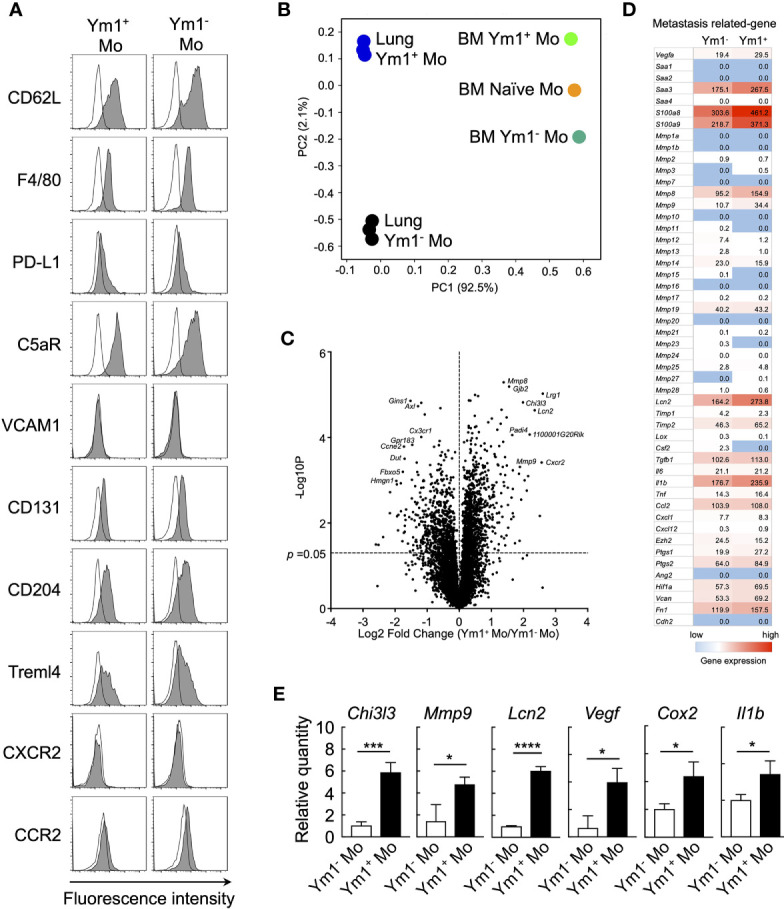
Ym1-Venus^+^Ly6C^hi^ monocyte express metastasis-related genes. **(A)** Ym1-Venus mice were injected intravenously with LPS (20 µg). Forty-eight hours later, the expression of surface markers of Ym1^+^ Mo and Ym1^-^ Mo in lung was analyzed by flow cytometry. Black lines indicate isotype control. **(B, D)** Gene expression profiles of Ym1^+^ Mo or Ym1^−^ Mo in lung and BM were globally compared by RNA-sequencing analysis. PCA **(B)**, volcano plots **(C)**, and heatmap of indicated genes **(D)**. **(E)** mRNA expression levels in Ym1^+^ Mo and Ym1^-^ Mo of lung. Ym1-Venus mice were injected intravenously with LPS (20 µg) followed by i.v. injection of B16 cells (1 x 10^5^ cells) 6 h later. Ym1^+^ Mo and Ym1^-^ Mo were sorted from BM of Ym1-Venus mice 48 h after LPS treatment. the expression of mRNA levels was analyzed. Average values are shown with SD. Unpaired two-tailed t-test, n = 3. ****P < 0.001; ***P < 0.005, *P < 0.05.

To elucidate the mechanisms underlying the promotion of metastasis by Ym1^+^Ly6C^hi^ monocytes, we sought to characterize the Ym1-Venus^+^Ly6C^hi^ monocyte subpopulation that accumulated in lung during systemic inflammation. Flow cytometry analysis revealed that the Ym1^+^Ly6C^hi^ monocyte subpopulation expressed the same levels of several monocyte surface markers as the Ym1^-^Ly6C^hi^ monocyte subpopulation (**Figure 3A**). Next, we globally compared the mRNA expression profiles of Ym1^+^Ly6C^hi^ monocytes and Ym1^-^Ly6C^hi^ monocytes from lungs of LPS-treated mice by RNA sequencing analysis. PCA analysis demonstrated that the two monocyte subsets exhibited obvious differences in gene expression after infiltrating into lung (**Figure 3B**). In addition, the gene expression of lung monocyte subsets clearly differed from previously reported that of BM monocyte subsets (22) (**Figure 3B**). While *Chi3l3* (Ym1-coding gene), known as a marker of M2 macrophages (34), is highly expressed in Ym1^+^Ly6C^hi^ monocytes, Ym1^+^Ly6C^hi^ monocytes did not show higher expression of any other M2 genes (**Supplemental Figure 7**). Interestingly, the expression of several metastasis-related genes (35) such as *Mmps*, *Vegf, Cox2*, and *Il1b* was enhanced in Ym1^+^Ly6C^hi^ monocytes, while expression levels of inflammatory cytokines except *Il1b* were not different between two subsets (**Figures 3C, D** and, **Supplemental Figure 7**). In addition to these genes, *Lcn2*, which is reported to enhance matrix metalloproteinase-9 (MMP-9) activity by stabilizing MMP-9 (36), was also expressed in higher levels in Ym1^+^Ly6C^hi^ monocytes. The high expression of those genes in Ym1^+^Ly6C^hi^ monocytes was also confirmed by PCR analysis (**Figure 3E**).

### MMP-9 Is Essential for Ly6C^hi^ Monocyte-Promoting Lung Metastasis

**Figure 4:**
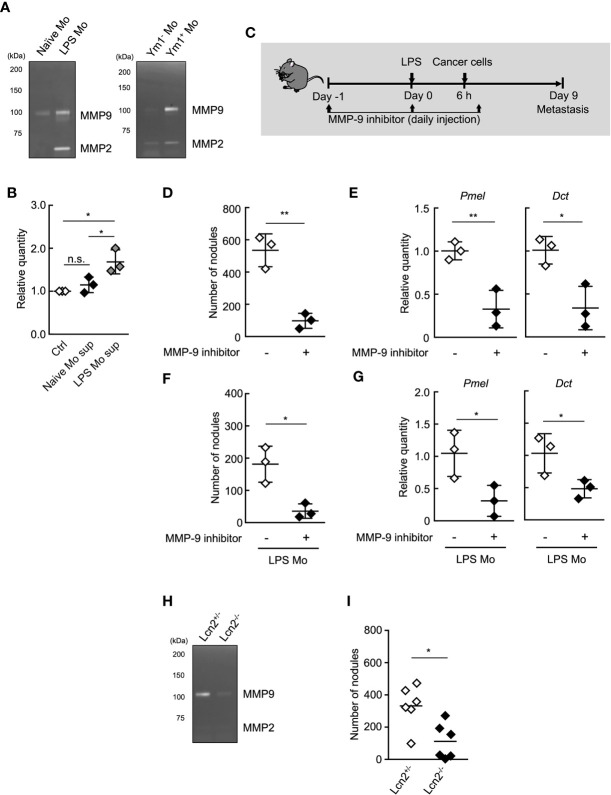
MMP-9 is essential for Ly6C^hi^ monocyte-promoting lung metastasis. **(A)** Gelatin zymography of culture supernatant of monocytes. Ly6C^hi^ monocytes isolated from naïve- or LPS-treated WT mice (left), or Ym1^+^ or Ym1^-^Ly6C^hi^ monocytes isolated from LPS-treated Ym1-Venus mice (right) were cultured for 24 h. The culture supernatants were assayed for gelatinase activity. Experiments were repeated twice with similar results. **(B)** Invasion assay of cancer cells in the presence of culture supernatant of Ly6C^hi^ monocytes isolated from naïve or LPS-treated WT mice. Average values are shown with SD. One-way ANOVA followed by Tukey’s test, n = 3. *P < 0.05; n.s., not significant. Each symbol represents an individual animal-derived culture supernatant. **(C–E)** Effects of MMP-9 inhibitor on LPS-promoted metastatic progression. **(C)** Experimental design used to test the effects of MMP-9 inhibitor injected at the early points of metastasis. WT mice were injected with 20 µg of LPS on Day 0 followed by i.v. injection of B16 cells (1 x 10^5^ cells) 6 h later. These mice were injected with 10% DMSO/PBS [inhibitor (-)] or MMP-9 inhibitor (SB-3CT, 250 µg) on Day -1, 0, 1. The lungs were analyzed for metastasis on Day 9. **(D)** Quantitative summary of the number of lung metastases. **(E)** mRNA expression levels of indicated genes in lungs are shown as fold change relative to control lungs. Average values are shown with SD. Unpaired two-tailed t-test, n = 3. **P < 0.01; *P < 0.05. Each symbol represents an individual animal. **(F, G)** Effects of MMP-9 inhibitor on metastasis promoted by LPS Mo transfer. WT mice were transferred intravenously with Ly6C^hi^ monocytes prepared from LPS-treated mice (LPS Mo) (5 x 10^5^ cells) on Day -1. Twenty-four hours later, B16 cells were injected intravenously. These mice were injected with 10% DMSO/PBS [inhibitor (-)] or MMP-9 inhibitor (SB-3CT, 250 µg) on Day -1, 0, 1. The lungs were analyzed for metastasis on Day 9. **(F)** Quantitative summary of the number of lung metastases on Day 9. **(G)** mRNA expression levels of indicated genes in lungs are shown as fold change relative to control lungs. Average values are shown with SD. Unpaired two-tailed t-test, n = 3. *P < 0.05; n.s., not significant. Each symbol represents an individual animal. **(H)** Gelatin zymography of culture supernatant of Lcn2^-/-^ monocytes. Ly6C^hi^ monocytes isolated from LPS-treated Lcn2^+/-^ or Lcn2^-/-^ mice were cultured for 24 h. The culture supernatants were assayed for gelatinase activity. Experiments were repeated twice with similar results. **(I)** Reduced number of metastatic foci in lung by the injection of Lcn2^-/-^ LPS Mo. LPS Mo were prepared from BM of Lcn2^+/-^ or Lcn2^-/-^ mice 48 h after LPS (20 µg) injection, and these monocytes were transferred intravenously into WT mice. Twenty-four hours later, B16 cells (1 x 10^5^ cells) were injected intravenously. The lungs were analyzed for metastasis on Day 9. Average values are shown. Unpaired two-tailed t-test, n = 6. *P < 0.05. Each symbol represents an individual animal.

MMP-9 plays an important role in the invasion and metastasis of cancer cells (37–40). Thus, we next sought to examine the roles of MMP-9 in metastasis promotion by Ym1^+^Ly6C^hi^ monocytes. We first examined the protein levels of MMP-9 in the culture supernatant of purified Ly6C^hi^ monocytes using gelatin zymography. The higher protein levels of proMMP-9, latent form of MMP-9 was observed in the culture supernatant of LPS Mo compared with Naïve Mo (**Figure 4A**). We also confirmed that Ym1^+^Ly6C^hi^ monocytes in LPS Mo showed higher protein levels of proMMP-9 than Ym1^-^Ly6C^hi^ monocytes in LPS Mo (**Figure 4A**). We next demonstrated whether the culture supernatant of LPS Mo promoted cancer cell invasion *in vitro* using the Matrigel invasion assay. The culture supernatant of LPS Mo induced a significant increase in cancer cell invasion compared with that of Naïve Mo (**Figure 4B**). We next demonstrated the *in vivo* contribution of Ym1^+^Ly6C^hi^ monocyte derived-MMP-9 to metastasis. The sequential injection of MMP-9 inhibitor suppressed LPS-promoted lung metastasis (**Supplemental Figure 8**). However, previous report suggested that MMP-9 induces not only the awakening of dormant cancer cells in lung through extracellular matrix remodeling (ECM) but also outgrowth of tumor growth in the late phase of metastasis (9). Thus, to inhibit *in vivo* MMP-9 enzymatic activity only at early time points, WT mice were injected with MMP-9 inhibitor on Days -1, 0, and 1 only (**Figure 4C**). Treatment of WT mice with the inhibitor at these time points also suppressed the number of metastatic foci (**Figures 4D, E**). The metastasis-promoting effects of injecting LPS Mo were also canceled by the early injection of the inhibitor (**Figures 4F, G**). We further tried to demonstrate that Ym1^+^Ly6C^hi^ monocyte-derived MMP-9 is responsible for the progression of metastasis by using lipocalin 2 (Lcn2) -deficient mice (27). As described above, Lcn2 is reported to be responsible for stability of MMP-9 (36). In fact, the protein levels of MMP-9 in Lcn2^-/-^ LPS Mo were lower than those in Lcn2^+/-^ LPS Mo (**Figure 4H**). The injection of Lcn2^-/-^ LPS Mo resulted in the reduced number of metastatic foci in lung compared with the case of Lcn2^+/-^ LPS Mo injection (**Figure 4I**). Taken together, these results indicate that Ym1^+^Ly6C^hi^ monocyte derived-MMP-9 has a strong impact on the promotion of lung metastasis.

### Ym1^+^Ly6C^hi^ Monocytes Contribute to the Promotion of Lung Metastasis Induced by Tumor Resection

**Figure 5:**
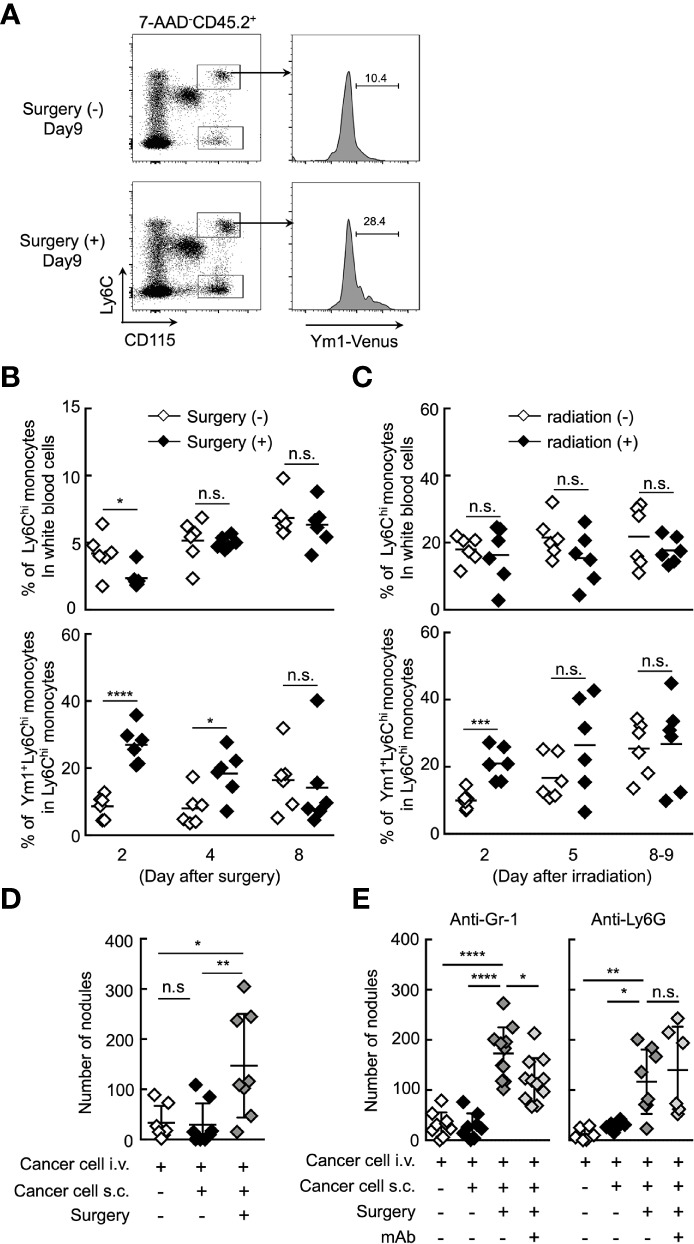
Ym1^+^Ly6C^hi^ monocytes contribute to the promotion of lung metastasis induced by tumor resection. **(A, B)** Increased frequency of Ym1^+^Ly6C^hi^ monocytes after tumor resection. B16 cells (1 x 10^6^ cells) were subcutaneously (s.c.) inoculated in the flank of Ym1-Venus mice. Seven days later, the primary tumor was removed by surgery. **(A)** The frequency of monocytes in peripheral blood were analyzed at the indicated time points. Representative flow cytometric profiles of the percentage of Ym1^+^ cells in Ly6C^hi^ monocytes on day 9. **(B)** Percentage of Ly6C^hi^ monocytes in white blood cells (upper) and Ym1^+^ cells in Ly6C^hi^ monocytes (lower) in peripheral blood. Unpaired two-tailed t-test, n = 5-6. ****P < 0.001; *P < 0.05; n.s., not significant. **(C)** Increased frequency of Ym1^+^Ly6C^hi^ monocytes after radiation exposure to primary tumor. B16 cells (1 x 10^6^ cells) were subcutaneously (s.c.) inoculated in the flank of Ym1-Venus mice. Seven to eight days later, the animals were randomized into one of two treatment groups: no irradiation or 30 Gy irradiation. The frequency of monocytes in peripheral blood were analyzed at the indicated time points. Percentage of Ly6C^hi^ monocytes in white blood cells (upper) and Ym1^+^ cells in Ly6C^hi^ monocytes (lower) in peripheral blood. Unpaired two-tailed t-test, n = 6. ***P < 0.005; n.s., not significant. Each symbol represents an individual animal. **(D)** Increased number of lung metastases after tumor resection. B16 cells (1 x 10^6^ cells) were s.c. inoculated in the flank of WT mice. Seven days later, the primary tumor was removed by surgery followed by i.v. injection of B16 cells (1 x 10^5^ cells) 24 h later. The lungs were analyzed for metastasis on Day 16. Quantitative summary of the number of lung metastases. Average values are shown with SD. One-way ANOVA followed by Dunnett’s test, n = 7-8. **P < 0.01; *P < 0.05; n.s., not significant. Each symbol represents an individual animal. **(E)** Effects of immune cell deletion on lung metastasis after tumor resection. B16 cells (1 x 10^6^ cells) were s.c. inoculated in the flank of WT mice. Seven days later, the primary tumor was removed by surgery followed by i.v. injection of B16 cells (1 x 10^5^ cells) 24 h later. For deletion of Ly6C^hi^ monocytes and neutrophils (anti-Gr-1), or neutrophils alone (anti-Ly6G), 50 µg/daily of indicated mAbs or PBS were injected into these mice from Day 6 to Day 15. The lungs were analyzed for metastasis on Day 16. Quantitative summary of the number of lung metastases. Average values are shown with SD. One-way ANOVA followed by Dunnett’s test, n = 6–11. ****P < 0.001; **P < 0.01; *P < 0.05; n.s., not significant. Each symbol represents an individual animal.

It was reported that the resection of primary tumor triggers a high frequency of tumor-dormancy escape and metastatic relapse in cancer (41–43). In mouse, inflammation associated with surgery triggered the outgrowth of distinct tumors or promoted metastasis (2, 7). Thus, we first sought to examine whether Ym1^+^Ly6C^hi^ monocytes are involved in the formation of metastatic foci after resection of tumors. B16 cells were inoculated subcutaneously. Subcutaneous tumors were removed by resection followed by i.v. injection of B16 cells 24 h after the resection. As shown in **Figures 5A, B**, the proportion of Ym1^+^Ly6C^hi^ monocytes, but not total Ly6C^hi^ monocytes, was drastically increased within 2 days after tumor resection. In addition to resection of primary tumor, it has already been reported that radiation exposure to primary tumor promotes cancer metastasis in mouse (44, 45). As expected, an increase in Ym1^+^Ly6C^hi^ monocytes was also observed in tumor-bearing mice treated with irradiation therapy (**Figure 5C**). Furthermore, we found that resection of the tumors promoted metastasis originating from CTCs (**Figure 5D**). We then tried to identify the immune cells responsible for the promotion of metastasis induced by resection. As shown in **Figure 5E**, anti-Ly6G mAb had no effects on metastasis. On the other hand, anti-Gr-1 mAb suppressed the metastatic formation. Taken together, we concluded that Ym1^+^Ly6C^hi^ monocytes contributed to the promotion of metastasis induced by tumor resection or radiation exposure to tumor.

### Inhibition of CXCR4 Signaling Reduces Lung Metastasis by Ym1^+^Ly6C^hi^ Monocytes

**Figure 6:**
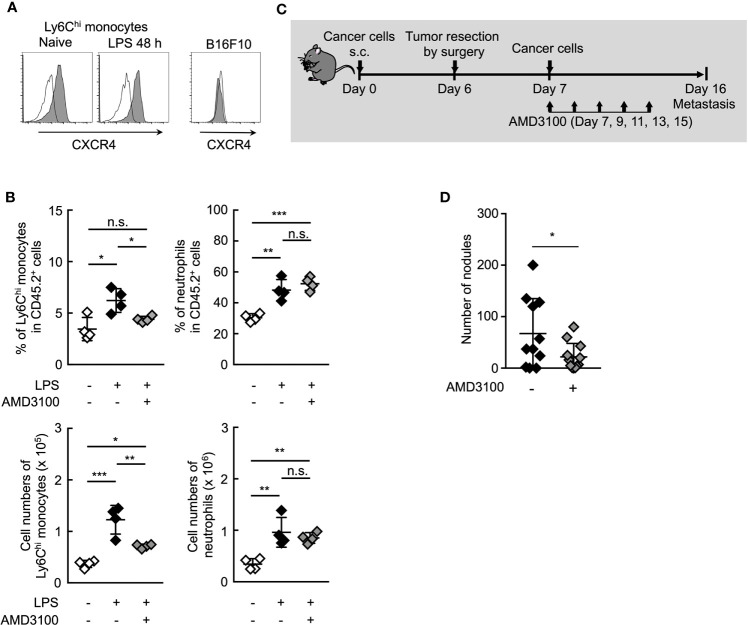
Inhibition of CXCR4 signaling reduce lung metastasis by Ym1^+^Ly6C^hi^ monocytes. **(A)** Expression of surface CXCR4 on peripheral blood Ly6C^hi^ monocytes isolated from naïve or LPS-treated mice, or B16 cells. Black lines indicate isotype control. **(B)** Inhibition of lung accumulation of Ly6C^hi^ monocytes by treatment with CXCR4 antagonist. WT mice injected intravenously with LPS (20 µg) and treated i.p. with AMD3100 (5 mg/kg, -1 h before and 24 h after LPS injection) or PBS [inhibitor (-)]. Average values are shown with SD. One-way ANOVA followed by Dunnett’s test, n = 4. ***P < 0.005; **P < 0.01; *P < 0.05; n.s., not significant. Each symbol represents an individual animal. **(C, D)** Effects of CXCR4 antagonist on lung metastasis after tumor resection. **(C)** Experimental design used to test the effect of AMD3100 treatment on tumor-resection-induced metastatic progression. B16 cells (1 x 10^6^ cells) were s.c. inoculated in the flank of WT mice. Six days later, the primary tumor was removed by surgery followed by i.v. injection of B16 cells (1 x 10^5^ cells) 24 h later. AMD3100 (5 mg/kg) or PBS [inhibitor (-)] was injected into these mice on Day 7, 9, 11, 13, and 15. The lungs were analyzed for metastasis on Day 16. **(D)** Quantitative summary of the number of lung metastases. Average values are shown with SD. Unpaired two-tailed t-test, n = 10–11. *P < 0.05. Each symbol represents an individual animal.

It is presumably critical for Ym1^+^Ly6C^hi^ monocytes to accumulate *in situ* for the promotion of lung metastasis. Chong et al. reported that the lung accumulation of Ly6C^hi^ monocytes is dependent on the CXCR4-CXCL12 signaling axis in the LPS-induced inflammation state (46). In fact, CXCR4 expression was observed on Ly6C^hi^ monocytes, but not B16 cells (**Figure 6A**). AMD3100, a CXCR4 antagonist, inhibited the accumulation of Ly6C^hi^ monocytes, but not neutrophils (**Figure 6B**) in lung associated with systemic inflammation. We then examined whether AMD3100 suppressed lung metastasis promoted by the surgical resection of primary tumor resection. The promotion of lung metastasis was inhibited by the treatment with AMD3100 (**Figures 6C, D**). These findings suggest that CXCR4 is a novel therapeutic target for controlling lung metastasis associated with surgical intervention for cancer by inhibiting the accumulation of Ym^+^Ly6C^hi^ monocytes in potential metastatic organs.

## Discussion

In this study, we demonstrated that Ym1^+^Ly6C^hi^ monocytes, but not neutrophils, promote inflammation-induced lung metastasis associated with intervention for primary tumor. Inhibition of the accumulation of Ym1^+^Ly6C^hi^ monocytes in lung or inhibition of MMP9 reduced lung metastasis, suggesting that Ym1^+^Ly6C^hi^ monocytes are a therapeutic target for the metastasis. Recent reports highlighted the critical roles of NETs in metastasis associated with inflammation in mouse. For instance, intranasal injection of LPS triggers marked neutrophil recruitment, detects numerous NET formation, and promotes lung metastasis (9). The induction of peritonitis results in NET formation in liver and facilitates liver metastasis (11). The inhibition of NET formation with DNase and neutrophil elastase inhibitor suppresses lung and liver metastases (11). In these experimental models, inflammation is elicited by local infection or tissue injury. However, such local inflammation at a distant site from the primary tumor dose not seems to occur in tumor-bearing patients. In this study, we demonstrated that a novel monocyte subset, but not neutrophils, play a critical role in the progression of metastasis associated with inflammation caused by primary tumor resection or irradiation. Although neutrophils were recruited into lung in this type of inflammation, NET formation hardly occurred at this site. These results indicate that the context of inflammation determines the types of immune cells primarily responsible for promoting metastasis, and suggest that therapeutic target cells for metastasis prevention need to be carefully selected according to the actual situation of cancer patients.

Recent reports have shown that a functionally distinct monocyte subset is differentiated in BM in response to certain inflammatory stimuli (16, 23, 25). This monocyte subset is differentiated from granulocyte-monocyte progenitors (GMPs) and shares some characteristics with granulocytes. In line with this concept, we showed in our previous report that immunoregulatory Ym1^+^Ly6C^hi^ monocytes were generated from GMPs in BM during the recovery phase of tissue injury and contributed to inflammatory response associated with tissue repair (22). It is well known that tissue repair and wound healing after tissue injury consist of multiple processes including regeneration of parenchymal cells, ECM remodeling and angiogenesis (47). These processes also contribute to tumor progression and metastasis (32). For example, the high expression of wound-response gene increased the risk of metastasis in human (48). Increased MMPs, which are critical molecules for ECM remodeling in injured tissue, are correlated with low overall survival rate in cancer patients (49). In fact, lung metastasis of B16F10 was decreased in MMP9-deficient mouse (40). Regarding the regeneration of blood vessels in wound tissue, the hypoxic condition is detected by hypoxia inducible factor alpha (HIF1a), which induces the production of vascular endothelial growth factor (VEGF) to promote angiogenesis (50). The same mechanisms also apply in cancer tissue, and neutralizing antibody against VEGF or VEGF receptor was reported to inhibit lung metastasis (51, 52). In this respect, the tissue repair process shares common features with cancer progression and metastasis. This study, together with our previous report, reveals a link between tissue repair and cancer progression from the perspective of cell population. In the future, the relationship between these monocytes and primary tumor progression should be investigated.

Patients who undergo resection of primary tumors face the risk of metastatic recurrence that peaks sharply 12 to 18 months after surgery (41–43). Although the cause of early metastatic relapse has been debated, a recent report has indicated that systemic inflammation induced by resection triggers the outgrowth of distant dormant tumors in mouse, implicating that Ly6C^hi^ monocytes are essential effector cells for the induction of the outgrowth (7). However, it is unclear what monocyte subset is involved in tumor progression induced by resection. In the present study, we showed that immunoregulatory Ym1^+^Ly6C^hi^ monocytes promoted lung metastasis of CTCs in an MMP-9- and CXCR4-dependent manner. MMP-9 secreted by Ym1^+^Ly6C^hi^ monocytes may degrade extracellular matrix and promote infiltration of cancer cells into metastatic tissues.

These results suggest that the risk of metastatic recurrence after resection can be reduced by developing a therapeutic method targeting immunoregulatory Ym1^+^Ly6C^hi^ monocytes.

The prediction and prevention of metastasis is a vital clinical task in cancer treatment. A previous report suggested that the high expression of wound response signature in tumors is a predictor of poor patient survival and increased risk of metastasis in human (48). In this study, we observed the rapid increase of Ym1^+^Ly6C^hi^ monocyte numbers in peripheral blood in the early stage of lung metastasis. Given that the emergence of Ym1^+^Ly6C^hi^ monocytes is implicated in the initial step of wound healing or tissue repair, monitoring of these monocytes in peripheral blood after surgical intervention for primary tumor may be a useful predictive cellular biomarker for metastasis. For this purpose, the human counterpart of mouse Ym1^+^Ly6C^hi^ monocytes should be identified. A recent study that employed single-cell RNA sequencing uncovered a four-monocyte population in healthy human peripheral blood (53). Furthermore, in cancer patients, monocytic myeloid-derived suppressor cells have emerged as the major negative regulators of immune responses (54–56). In any case, estimation of the emergence of novel and atypical monocyte subsets associated with inflammation is important for the development of therapeutic strategies for metastasis.

## Data Availability Statement

The RNA-seq data has been deposited to the GEO - accession number is GSE174199.

## Ethics Statement

The animal study was reviewed and approved by Tokyo University of Pharmacy and Life Sciences Animal Use Committee.

## Author Contributions

TS: Data curation, Formal analysis, Validation, Investigation, Methodology. AK: Validation, Investigation. HS: Validation, Investigation. KK: Data curation, Funding acquisition. MM: Validation, Investigation. RS: Validation, Investigation. NI: Conceptualization. KA: Funding acquisition, Writing-review and editing. DK: Data curation, Investigation. TT: Data curation, Investigation. AY: Resources. KI: Resources. TSa: Resources. SA: Resources. MT: Conceptualization, Supervision, Funding acquisition, Methodology, Writing-original draft, Project administration, Writing-review and editing. SY: Conceptualization, Data curation, Formal analysis, Supervision, Funding acquisition, Validation, Investigation, Methodology, Writing-original draft, Writing-review and editing. All authors contributed to the article and approved the submitted version.

## Conflict of Interest

The authors declare that the research was conducted in the absence of any commercial or financial relationships that could be construed as a potential conflict of interest.
