# Supplementary material for: Immunoregulatory Monocyte Subset Promotes Metastasis Associated With Therapeutic Intervention for Primary Tumor
Source: Front Immunol. 2021 Jun 7;12:663115. doi: 10.3389/fimmu.2021.663115 (PMC8215602; doi:10.3389/fimmu.2021.663115)
Supplement: Supplementary file 1 [file DataSheet_1.pdf]

Supplemental Figure 1

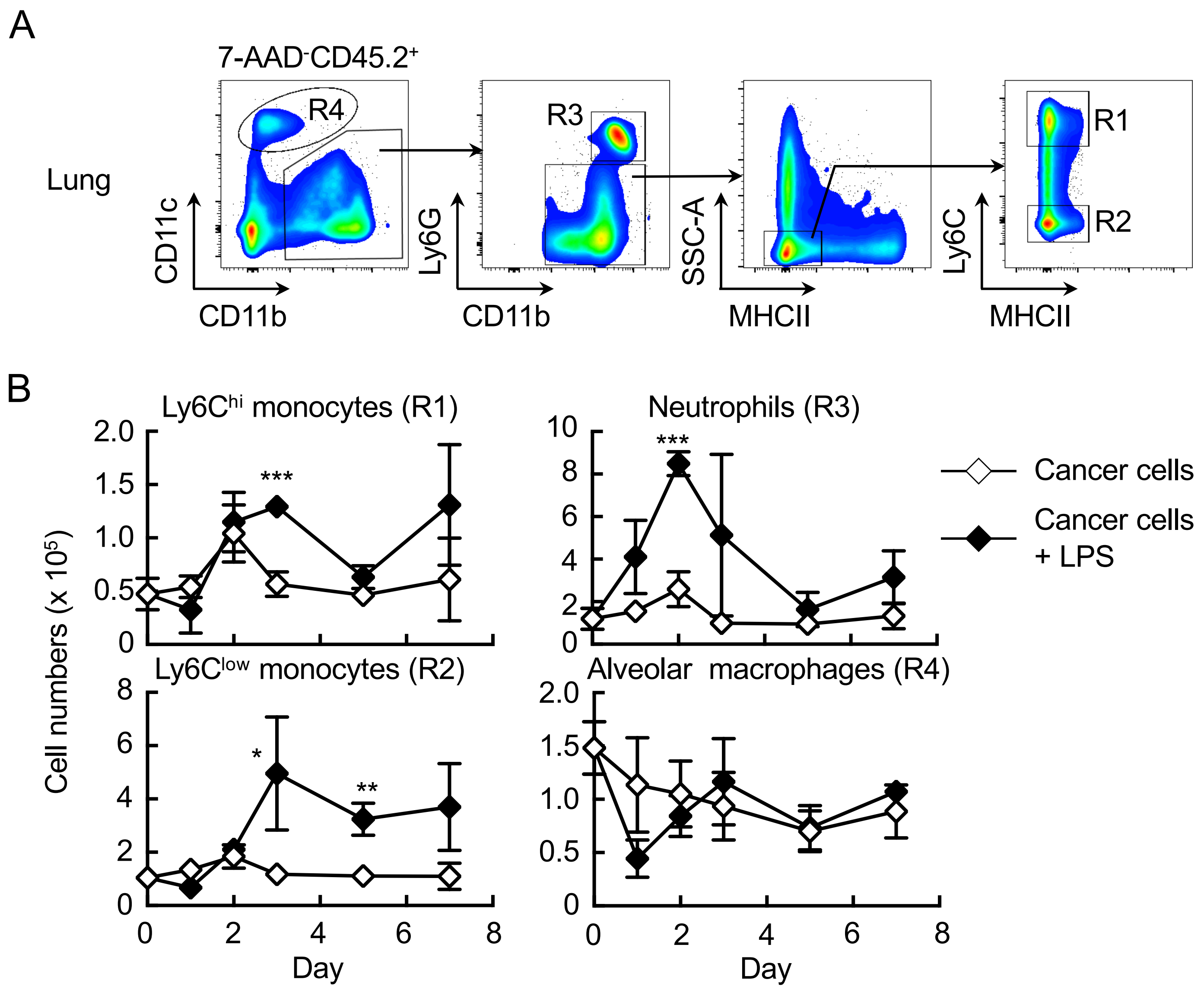

Supplemental Figure 1. Identification of immune cells in lung by flow cytometry. (A) Gating strategy for lung immune cells. Samples were pregated on live CD45.2^{+} cells. To identify Ly6C^{hi} monocytes (R1), Ly6C^{low} monocytes (R2), neutrophils (R3), and alveolar macrophages (R4), Ly6C, Ly6G, MHCII, CD11b, and CD11c expression was assessed by flow cytometry. Representative analysis of lung immune cells from PBS-injected Ym1-Venus mice are shown. **(B)** Absolute numbers of immune cells in lungs of LPS-treated mice. WT mice were injected intravenously (i.v.) with LPS (20 μg) followed by i.v. injection of B16 cells (1x10^{5} cells) 6 h later. At the indicated time points, the number of immune cells in lungs was counted by flow cytometry. Average values are shown with SD. Unpaired two-tailed t-test at each time point, compared with cancer cell injection, n = 3-4. ***$P <$ 0.005; **$P < 0.01$; *$P < 0.05$.

T. Shibuya et al.

Supplemental Figure 2

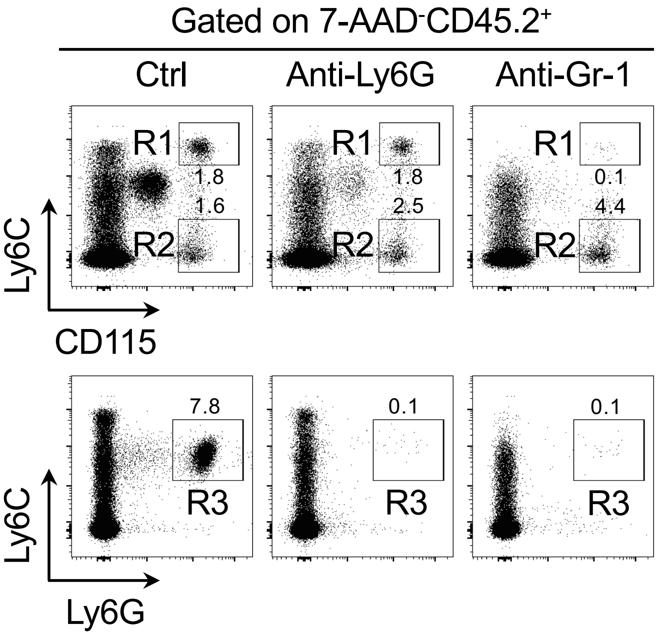

Supplemental Figure 2. Flow cytometric analysis of depletion-antibody-treated WT mouse. WT mice were injected intraperitoneally (i.p.) with PBS (Ctrl), anti-Ly6G mAb or anti-Gr-1 mAb (50 µg). Twenty-four hours later, to identify Ly6C^{hi} monocytes (R1), Ly6C^{low} monocytes (R2), and neutrophils (R3) in peripheral blood, Ly6C, CD115, and Ly6G expression was assessed by flow cytometry.

T. Shibuya et al.

Supplemental Figure 3

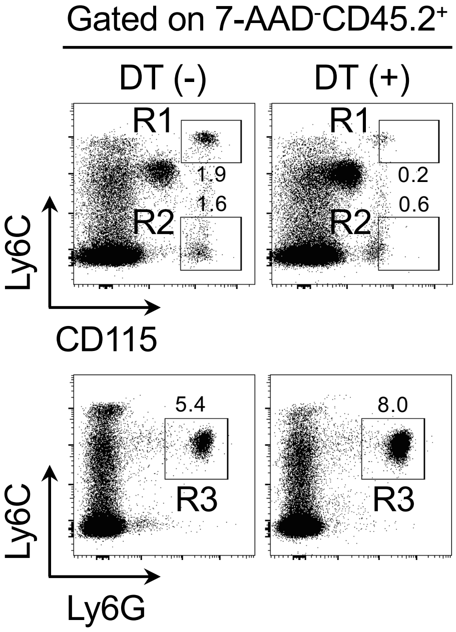

Supplemental Figure 3. Flow cytometric analysis of peripheral blood from CD204-DTR mice. CD204-DTR mice were injected i.p. with DT (500 ng). Twenty-four hours later, Ly6C^{hi} monocytes (R1), Ly6C^{low} monocytes (R2), and neutrophils (R3) in peripheral blood were identified by flow cytometry.

T. Shibuya et al.

Supplemental Figure 4

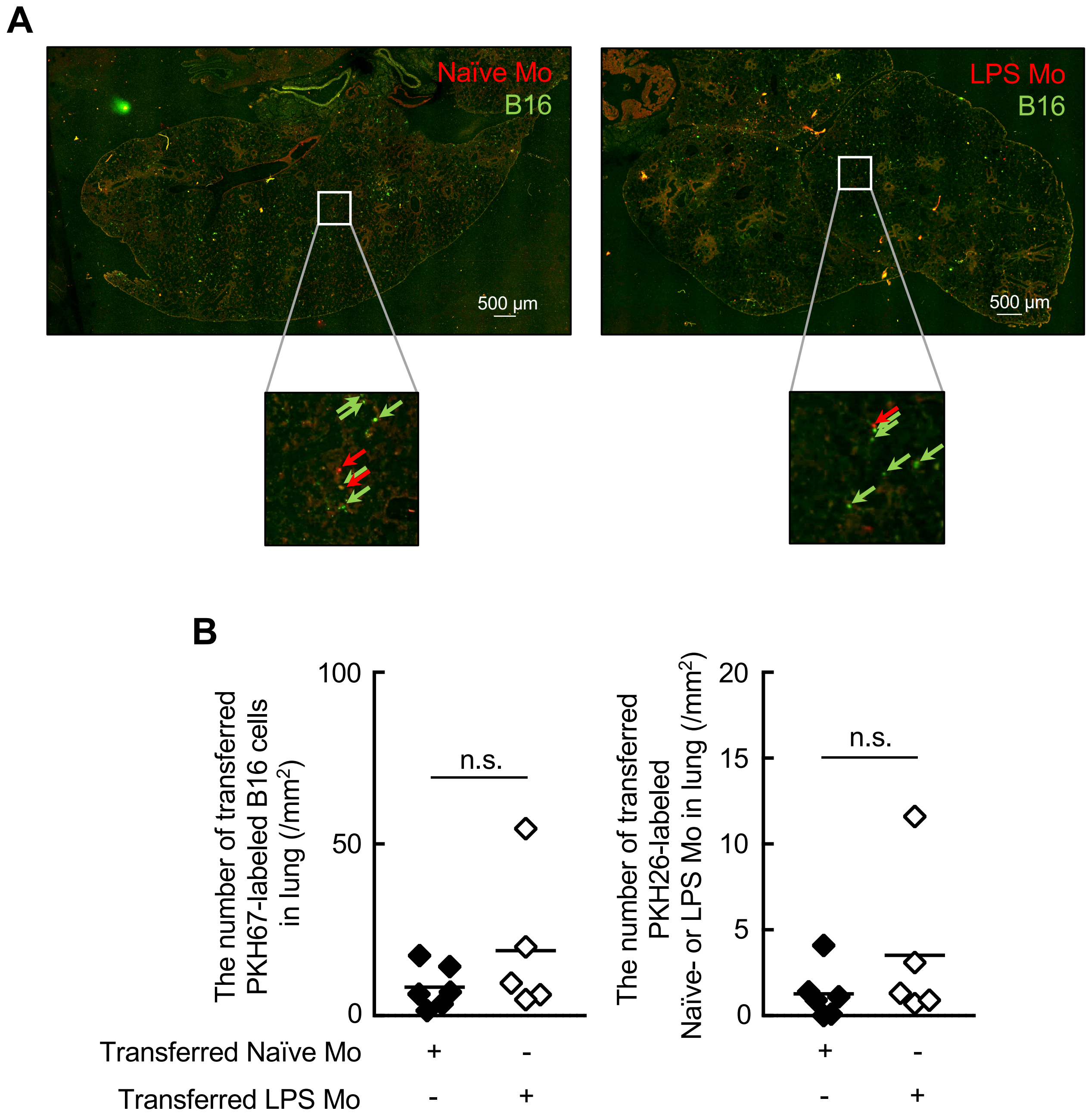

Supplemental Figure 4. The number of naïve or LPS Mo and B16 cells in lung. (A) Distribution of transferred monocytes (red arrow) and tumor cells (green arrow) in lung. WT mice were injected i.v. with PKH26-labeled Naïve Mo or LPS Mo (1 x 10^6 cells). After 90 min, WT mice were injected i.v. with PKH67-labeled B16 cells (1 x 10^6 cells). After another 90 min, cryosection of the lungs were observed under a fluorescence microscopy. Original magnification, x10. **(B)** The number of B16 cells (left), transferred Naïve Mo, and LPS Mo (right) in lung of (A). Unpaired two-tailed t-test, n = 5-6, n.s., not significant. Each symbol represents an individual animal.

Supplemental Figure 5

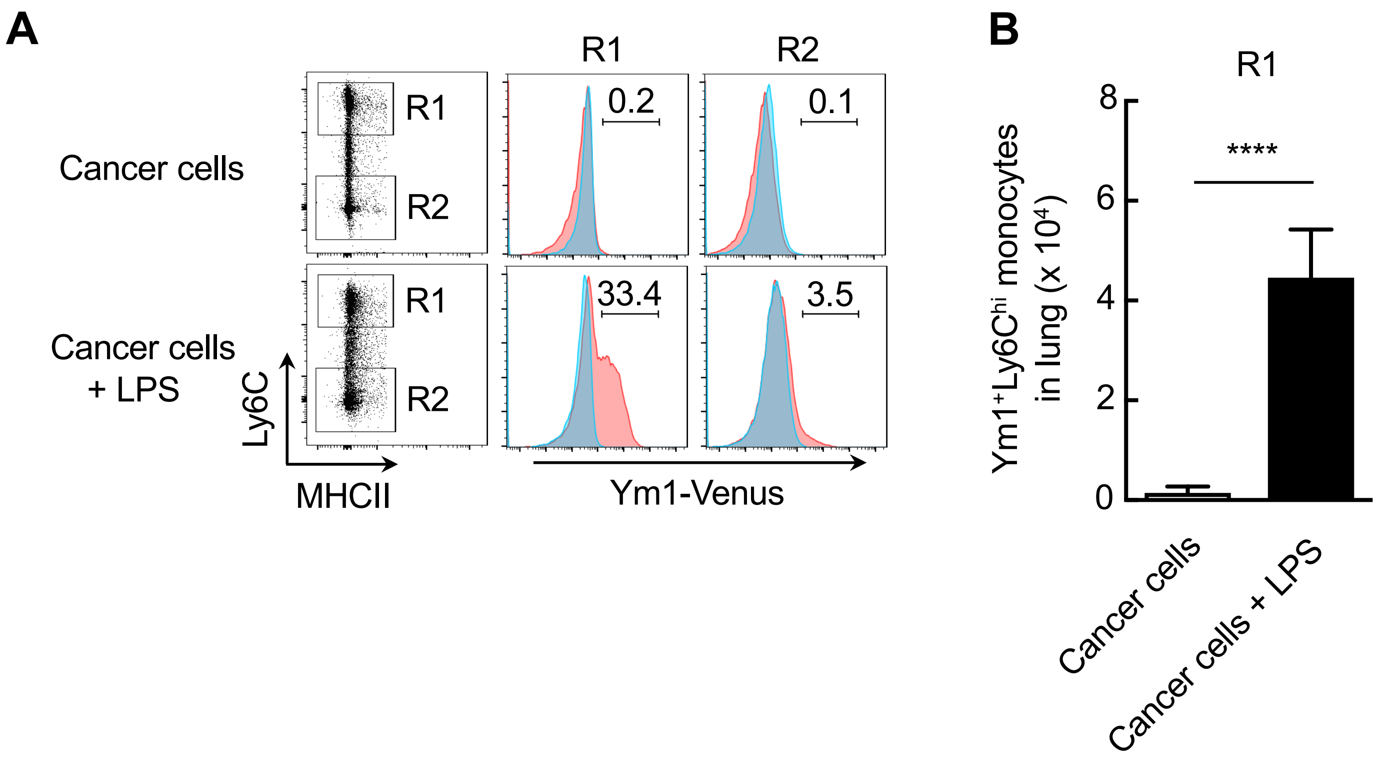

Supplemental Figure 5. Flow cytometric analysis of lung cells in WT (shaded area in blue) or Ym1-Venus mouse (in red) injected with cancer cells with or without LPS. (A) LPS was injected i.v. into WT or Ym1-Venus mice, and this was followed by the i.v. injection of B16 cells. Forty-eight hour later, lung cells were stained as described in Supplemental Figure1A. Numbers indicated percentage of $Ym1^{+}$ cells in $CD45.2^{+}CD11c^{-}CD11b^{+}MHCII^{-}Ly6C^{hi}$cells (R1; $Ly6C^{hi}$ monocytes, R2; $Ly6C^{low}$ monocytes). **(B)** Absolute number of Ym1-$Venus^{+}Ly6C^{hi}$ monocytes in lungs of (G). Average values are shown with SD. Unpaired two-tailed t-test, n = 5-7. ****$P <$ 0.001.

T. Shibuya et al.

Supplemental Figure 6

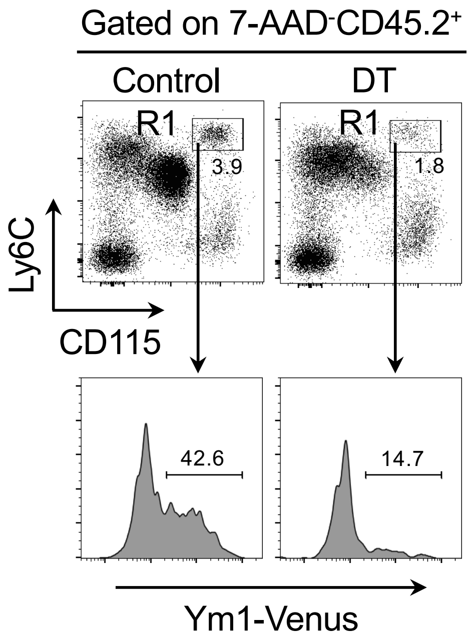

Supplemental Figure 6. Flow cytometric analysis of DT-treated Ym1-DTR-Venus mouse. Ym1-DTR-Venus mice were injected i.p. with DT. Forty-eight hours later, Ly6C^{hi} monocytes (R1) in peripheral blood were analyzed by flow cytometry. Note that DT injection specifically deletes Ym1-Venus^{+}Ly6C^{hi} monocytes.

T. Shibuya et al.

Supplemental Figure 7

Cytokines

	Ym1$^-$	Ym1$^+$
Il1a	8.7	13.6
Il1b	176.7	235.9
Il1f5	0.0	0.0
Il1f6	0.0	0.1
Il1f8	0.0	0.0
Il1f9	9.5	22.8
Il1f10	0.0	0.0
Il2	0.0	0.0
Il20	0.0	0.0
Il3	0.0	0.0
Il4	0.7	0.0
Il4i1,Nup62,Nup62-il4i1	38.1	29.0
Il5	0.0	0.0
Il6	21.1	21.2
Il6st	29.6	21.2
Il7	1.0	0.9
Il9	0.0	0.0
Il10	2.4	4.4
Il11	0.2	0.3
Il12a	1.0	0.7
Il12b	0.7	1.0
Il13	0.0	0.0
Il15	28.1	33.5
Il16	46.6	46.6
Il17a	0.0	0.1
Il17b	0.0	0.0
Il17c	0.0	0.0
Il17d	0.0	0.0
Il17f	0.0	0.0
Il18	88.3	94.7
Il18bp	9.6	20.9
Il19	0.1	0.1
Il21	0.0	0.0
Il22	0.0	0.0
Il23a	0.0	0.6
Il24	0.0	0.0
Il25	0.0	0.0
Il27	13.5	21.0
Il28a	0.0	0.0
Il28b	0.0	0.0
Il31	0.0	0.0
Il33	0.3	0.0
Il34	0.0	0.0

M1 gene

	Ym1$^-$	Ym1$^+$
Il6	21.1	21.2
Il1b	176.7	235.9
Nos2	1.5	4.5
Cxcl1	7.7	8.3
Cxcl2	70.1	107.3
Il12a	1.0	0.7

M2 gene

	Ym1$^-$	Ym1$^+$
Chi3l3	169.7	239.8
Il10	2.4	4.4
Arg1	6.1	17.1
Mrc1	11.6	5.5
Msr1	121.8	148.8
Retnla	1.5	6.1

low high

Gene expression

Supplemental Figure 7. Gene expression profiles of Ym1$^+$Ly6C^{hi} monocytes or Ym1$^-$Ly6C^{hi} monocytes from LPS-injected Ym1-Venus mice were globally compared by RNA-seq analysis.

Supplemental Figure 8

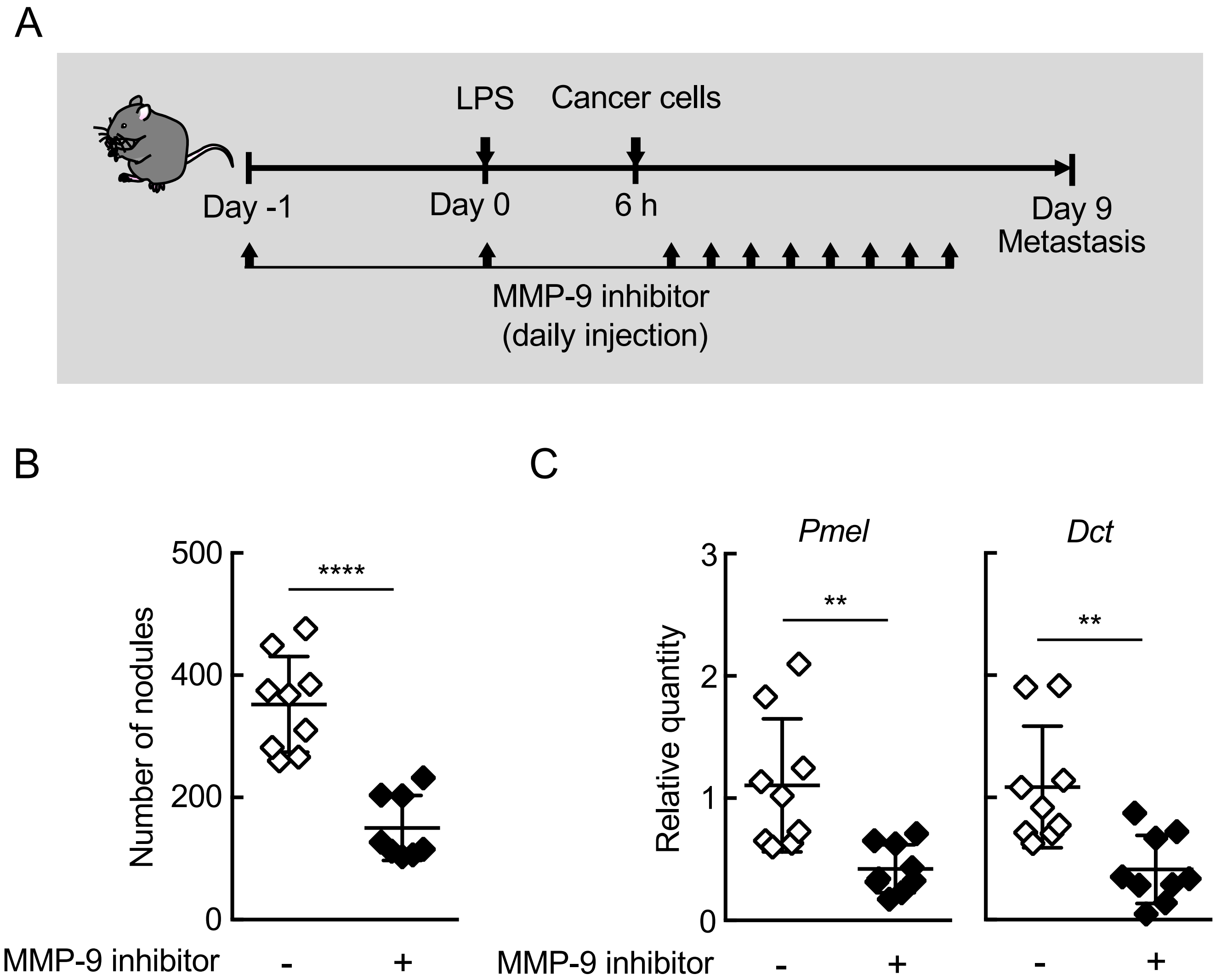

Supplemental Figure 8. Sequential injection of MMP-9 inhibitor suppresses LPS-promoted lung metastasis. (A) Experimental design used to test the effects of sequential injection of MMP-9 inhibitor. WT mice were injected with 20 μg of LPS on Day 0 followed by i.v. injection of B16 cells (1 x 10^5 cells) 6 h later. These mice were injected with 10% DMSO/PBS (inhibitor (-)) or MMP-9 inhibitor (SB-3CT, 250 μg) once daily for Day -1 to 8. The lungs were analyzed for metastasis on Day 9. **(B)** Quantitative summary of the number of lung metastases. **(C)** mRNA expression levels in lungs of (B). mRNA expression levels of indicated genes were determined by qRT-PCR and are shown as fold change relative to control lungs. Average values are shown with SD. Unpaired two-tailed t-test, n = 8-9, ****$P < 0.001$; **$P < 0.01$. Each symbol represents an individual animal.

T. Shibuya et al.
